# Research status and trends of the diabetic cardiomyopathy in the past 10 years (2012–2021): A bibliometric analysis

**DOI:** 10.3389/fcvm.2022.1018841

**Published:** 2022-10-20

**Authors:** Sicheng Wang, Chuanxi Tian, Zezheng Gao, Boxun Zhang, Linhua Zhao

**Affiliations:** ^1^Institute of Metabolic Diseases, Guang’anmen Hospital, China Academy of Chinese Medical Sciences, Beijing, China; ^2^Graduate School, Beijing University of Chinese Medicine, Beijing, China

**Keywords:** diabetic cardiomyopathy, cardiomyopathy, diabetic complications, bibliometric analysis, data visualization

## Abstract

**Background:**

Diabetic cardiomyopathy is one of the most life-threatening diabetic complications. However, the previous studies only discuss a particular aspect or characteristic of DCM, the current state and trends were explored by limited research. We aimed to perform a systemically bibliometric study of DCM research progress status in the past decade, visualize the internal conceptual structure and potential associations, and further explore the prospective study trends.

**Methods:**

Articles related to DCM published from January 2012 to December 2021 were collected in the Web of Science core collection (WoSCC) database on June 24, 2022. We exported all bibliographic records, including titles, abstracts, keywords, authorship, institutions, addresses, publishing sources, references, citation times, and year of publication. In addition, the journal Impact Factor and Hirsch index were obtained from the Journal Citation Report. We conducted the data screening, statistical analysis, and visualization *via* the Bibliometrix R package. VOS viewer software was employed to generate the collaboration network map among countries and institutions for better performance in visualization.

**Results:**

In total, 1,887 original research articles from 2012 to 2021 were identified. The number of annual publications rapidly increased from 107 to 278, and a drastic increase in citation times was observed in 2017–2019. As for global contributions, the United States was the most influential country with the highest international collaboration, while China was the most productive country. Professor Cai Lu was the most prolific author. Shandong University published the most articles. *Cardiovascular Diabetology* journal released the most DCM-related articles. “Metabolic Stress-induced Activation of FoxO1 Triggers Diabetic Cardiomyopathy in Mice” Battiprolu PK et al., J Clin Invest, 2012. was the most top-cited article regarding local citations. The top three keywords in terms of frequency were apoptosis, oxidative stress, and fibrosis. The analysis of future topic trends indicated that “Forkhead box protein O1,” “Heart failure with preserved ejection fraction,” “Dapagliflozin,” “Thioredoxin,” “Mitochondria dysfunction,” “Glucose,” “Pyroptosis,” “Cardiac fibroblast” and “Long non-coding RNA” could be promising hotspots.

**Conclusion:**

This study provides meaningful insights into DCM, which is expected to assist cardiologists and endocrinologists in exploring frontiers and future research directions in the domain through a refined and concise summary.

## Introduction

Diabetes mellitus (DM) is a prevalent chronic non-communicable disease and one of the most severe and pressing health issues worldwide. Globally, the number of diabetic patients has increased sharply in recent years, it is estimated that 537 million people live with DM worldwide in 2021, which will soar to 784 million in 2045 according to current estimates from International Diabetes Federation (IDF) Diabetes Atlas (1). Cardiovascular disease (CVD) is the leading cause of death in patients with DM, and the association between DM and CVD has been demonstrated for a long time (2, 3). However, several clinical observations including the Framingham Study found that there is a high risk of heart failure (HF) in patients with DM, ranging from 19% to 26% (4–6), and it even occurs independently of traditional CVD risk factors, including coronary heart disease (CAD), hypertension (HTN), etc. In 1972, Rubler and his colleagues observed a unique myocardial injury, and named it the diabetic cardiomyopathy (DCM) (7).

DCM initially has a subclinical period that results from fibrosis, left ventricular hypertrophy (LVH), and myocardial relaxation abnormality manifesting as asymptomatic in early phases (8). Still, with the progress of the disease, the growth of left ventricular mass (LVM) leads to the decline of diastolic left ventricular filling, it gradually becomes symptomatic from diastolic dysfunction to systolic dysfunction, manifesting as cardiac dysfunction eventually characterized by various metabolic and neurohumoral pathway disorders (9–11). Studies have shown that the prevalence of cardiac dysfunction in individuals with type 1 diabetes mellitus (T1DM) and type 2 diabetes mellitus (T2DM) is 14.5% and 35%, respectively (12, 13). Due to the insidious nature of DCM onset, the incidence of DCM has been dramatically underestimated (11, 14), meaning that the number of people with diabetes and cardiac dysfunction is far more significant than expected. DCM research is, therefore, of paramount importance in reducing the global healthcare burden and mortality among patients with DM. Although numerous reviews have previously addressed DCM from pathophysiology, preclinical and clinical perspectives, the previous reviews only discuss a particular aspect or characteristic of DCM through subjectively papers summary, conclusion, and extraction by researchers. Up to now, there is no study to comprehensively present the current status of DCM research including scholars, institutions, countries, journals, and research hotspots. Moreover, the traditional review is difficult to visualize the internal conceptual structure and potential associations of abundant literature objectively.

Bibliometrics analysis, proposed in 1969 by Pritchard, is a quantitative science approach that evaluates the research characteristics and trends in a specific time frame through many published academic literature analyses (15). Compared with traditional systemic reviews, it can be used not only to trace the historical evolution of a particular field, but also to predict the future research directions and collaboration opportunities through visualizing the conceptual, intellectual, and social structures at different scales from macro and micro perspectives. In recent years, with the emergence of an extensive number of medical academic publications, bibliometrics has played a vital role in the health care field. Due to the lack of tools to describe and analyze a massive amount of literature previously, bibliometrics analysis has not yet been used to systematically summarize the literature on DCM. Nowadays, multiple scientometric visualization software are available for bibliometrics analysis. The R programing language is an open-source software with robust analysis and visualization capabilities. As an R-package, *Bibliometrix* is a widely used science mapping application (16), and Java-run software VOS viewer (17) performs better in co-occurrence network visualization, which can help us track frontier dynamics by exploring core items in relevant research fields.

In this study, we retrieved DCM-related publications in the Web of Science™ core collection database over the past decade from 2012 to 2021, conducted an informative systematic and scientific overview, and predicted potential development trends by the following steps: (1) Investigate the output and growth trends of publications and citations; (2) Describe the distribution and characteristic of core countries, authors, institutions, journals, and top-cited publications; (3) Visualize the collaboration and co-occurrence between core countries, authors, and institutions; (4) Conduct a network of core author’s keywords and predict research hotspots and trends by algorithms. Overall, we aim to summarize issues in the literature review on DCM in the past decade and predict future trends. The findings will help academics, including cardiologists and endocrinologists gain a quick understanding of the current state of the DCM knowledge domain in the past decade and help them select journals for publication and collaborators, as well as guide directions and lay solid foundations for future studies.

## Materials and methods

### Dataset establishment

The online DCM literature data are publications collected from the Science Citation Index Expanded (SCIE) of Clarivate Analytics’ Web of Science^TM1^ core collection (WoS; WoSCC) database. As a well-known authoritative citation index database of research publications and citations (18), The WoSCC is one of the top sources for bibliometric analysis with a well-established citation network in different research fields, including natural sciences, engineering, biomedicine, etc., (19, 20). To understand the research status in the field of DCM in the past decade, we designed a search strategy to establish our dataset initially: [TS = (diabetic NEAR/0 myocardial) OR TS = (diabetic NEAR/0 cardiomyopathy) OR TS = (diabetic NEAR/0 myocardiopathy) OR TS = (diabetic NEAR/0 cardiomyopathies)] AND PY = (2012.1.1–2021.12.31). We excluded publications in the year of 2022 to obtain a more accurate annual result. All bibliographic records, including titles, abstracts, keywords, authors, institutions, addresses, journals, references, citation times, publication year, etc., were saved as plain TXT files. Our study data were obtained from an open database, so there are no ethical concerns.

### Data screening

**FIGURE 1:**
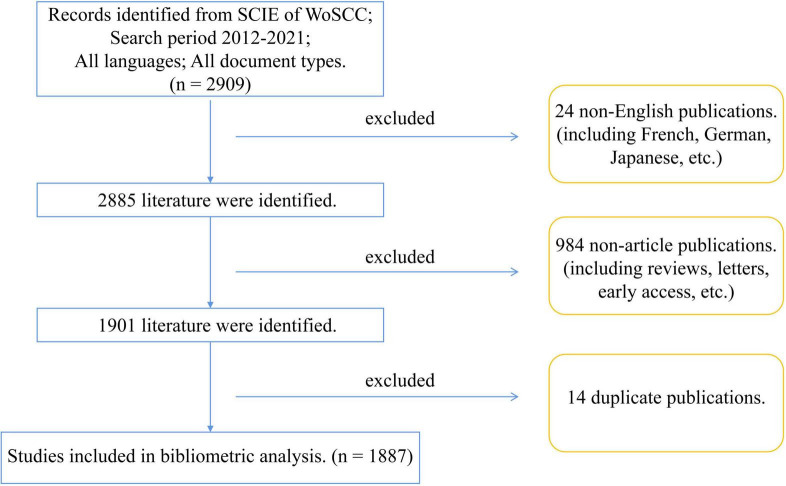
Flow chart of the data identification and screening results.

We integrated all txt files into a zip package and imported them into the analysis software *Biblioshiny* for data screening, which is a partner web interface app version of the Bibliometric R package *Bibliometrix* version 3.2.1 (R version 4.2.0, R studio version 2022.02.2 + 485 “Prairie Trillium” Release) makes the command line function more intuitive and user-friendly (16). To more precisely perform the research status in the field of DCM in the past decade, we used *Biblioshiny* to screen only full-length original articles that meet the requirement of our study, and non-article publications were excluded. The language was restricted to English. Moreover, we conducted a string of codes by *Readxl* R package version 1.4.0 (R version 4.2.0, R studio version 2022.02.2 + 485 “Prairie Trillium” Release) to identify and delete duplicate publications. The detailed search and screen procedure is shown in Figure 1. To avoid bias caused by database updates and subjectivity, two authors (WSC and TCX) independently performed data identification and screening on June 24, 2022. The third author (ZBX) made judgments of discrepancies to reach a consensus. Finally, our DCM research status dataset was exported as a CSV file.

### Data processing

We imported the CSV file for further data processing in the *Biblioshiny* R package, which can automatically analyze all the selected records and generate relative graphs with a mouse click. In our study, the *Biblioshiny* was employed to analyze all publication characteristics, including publication and citation trends, contributions of authors and journals, collaborations of institutions and countries, and distribution and prediction of hotspots, respectively. To perform more vivid and comprehensible collaboration maps, VOS viewer (version 1.6.18.0, Leiden University, Netherlands), which is one of the most widely used visualization software in bibliometric analysis (17), was employed to visualize collaboration maps among institutions and countries respectively, according to the PageRank score calculated from the *Biblioshiny* of R studio software interface.

It is worth mentioning that we assessed the journal impact factor (IF) and journal citation reports (JCR) (^2^ Clarivate Analytics, Philadelphia, United States) category according to the 2021 JCR™. The Hirsch index (H-index) indicates the academic influence of authors, that is, an author has published at most h papers that have been cited at least h times (21). Local citations (LCS) are used to evaluate the number of times a journal included in this dataset is cited by other journals in the same data set (22). The PageRank algorithm was invented to catalog the Internet web page by Larry Page, the Google company sponsor, becoming a popular and newly emerged bibliometric method for network citation analysis based on the structural characteristics of publications nowadays (23–25). Google used it to embody the relevance and importance of different web pages (26). Now, as an alternative measurement of impact for authors, intuitions, journals, etc., PageRank can sort nodes (Countries and institutions, in our study) by importance (PageRank score) which depends on the number of being cited and the score of each citing items themselves (27).

## Results

### Publication and citation trends in the past decade

**FIGURE 2:**
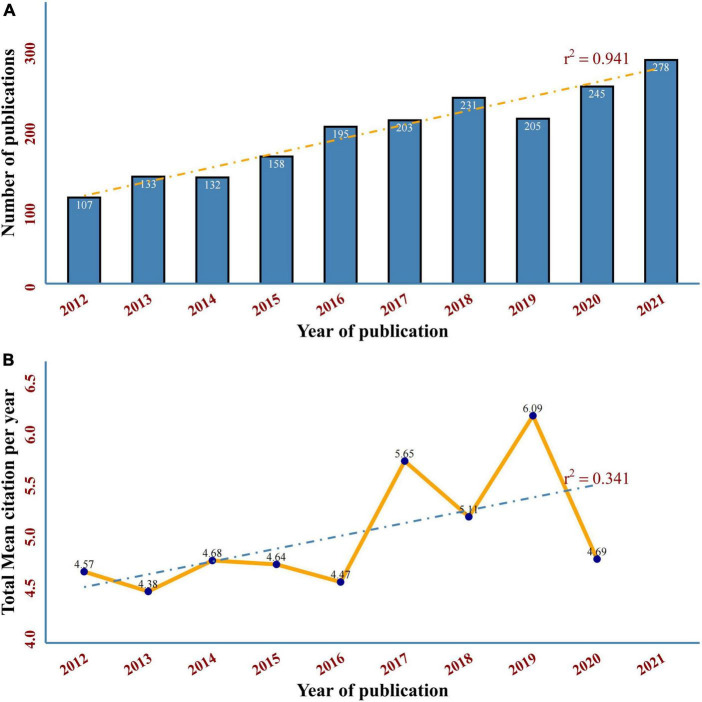
**(A)** Annual trend chart of publications on DCM in 2012–2021. The dotted line represents linear growth with r^2^ = 0.941. **(B)** Growth of the article cited per year in DCM. The dotted line represents linear growth with r^2^ = 0.341.

As shown in Figure 2A. The number of annual publications rapidly increased from 107 to 278 in the past decade, with more than two times the number of publications in 2021 than in 2012, indicating that DCM is an emerging focus of diabetes-related diseases. The total mean citation per year means the yearly average number of times each DCM disease-related publication has been cited. Interestingly, a drastic increase in citation times was observed in 2017–2019, reaching a peak of 6.09 in 2019 (Figure 2B).

### Analysis of countries and institutions

Table 1 lists the top 10 collaborating countries and institutions in the DCM research field in the past decade ranked by PageRank value and the number of publications—the greater the PageRank value, the more significant its importance, which represents it has more weight in cooperation. The top 50 countries and institutions ranked by PageRank are taken for visual analysis *via* VOS viewer. Circles represent different countries or institutions, the circle size represents the PageRank value, the lines signify the countries’ or institutions’ collaboration strength, and each color represents a cluster, which is a group of items with comparable attributes within a network. As we expected, related countries and institutions had multi-dimensional cooperation in the field of DCM.

**TABLE 1:** Top 10 countries and institutions in terms of PageRank and publications.

Items	PageRank			Publications		
	Ranking	Name	Value	Ranking	Name	Number
Countries	1	United States	0.196	1	China	977
	2	China	0.144	2	United States	235
	3	United Kingdom	0.056	3	Canada	72
	4	Canada	0.054	4	India	59
	5	Italy	0.053	5	Japan	43
	6	Germany	0.05	6	Australia	42
	7	Australia	0.042	7	Germany	40
	8	Japan	0.027	8	Italy	40
	9	Saudi Arabia	0.025	9	Egypt	36
	10	Egypt	0.025	10	Korea	27
Institutions	1	Wenzhou Med Univ	0.068	1	Shandong Univ	160
	2	Univ Louisville	0.061	2	Wenzhou Med Univ	144
	3	Jilin Univ	0.057	3	Harbin Med Univ	143
	4	Univ Melbourne	0.057	4	Univ Louisville	114
	5	Univ Hong Kong	0.046	5	Jilin Univ	105
	6	Monash Univ	0.044	6	Fourth Mil Med Univ	97
	7	Soochow Univ	0.039	7	China Med Univ	89
	8	Univ Alabama Birmingham	0.035	8	Huazhong Univ Sci And Technol	71
	9	Huazhong Univ Sci And Technol	0.033	9	WUHAN UNIV	69
	10	Baker Heart And Diabet Inst	0.033	10	Univ Melbourne	57

**FIGURE 3:**
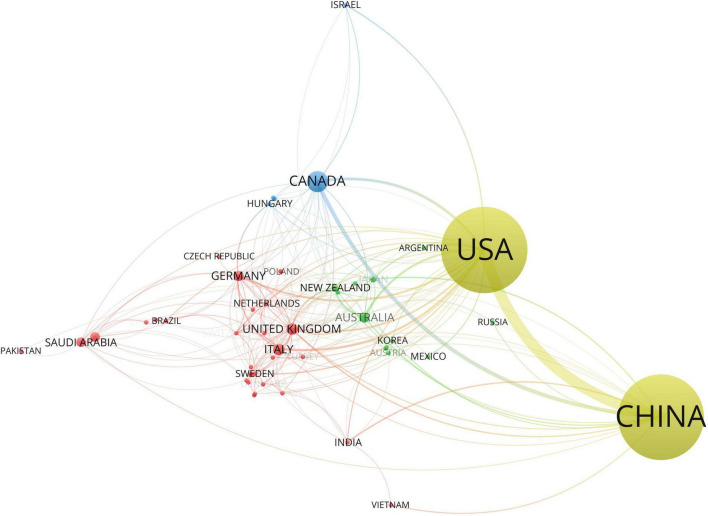
Top 50 Countries’ collaboration network map in the field of DCM ranked by PageRank.

**FIGURE 4:**
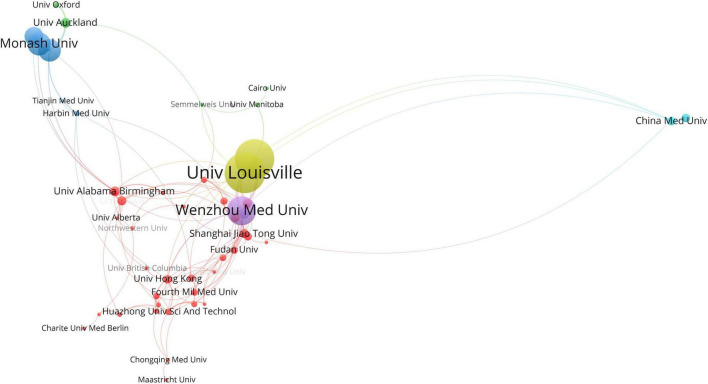
Top 50 Institutions’ collaboration network map in the field of DCM ranked by PageRank.

In Figure 3, the United States (Publications: 235, RankPage: 0.196) ranks first in the PageRank value, connected to almost all countries in the figure, and China (Publications: 977, RankPage: 0.144) ranks first in the total number of publications and has the most robust connection with the United States. Figure 4 shows the institutions’ cooperation network. The top 5 collaborating institutions in the past decade are Wenzhou Medicine University (Publications: 144, RankPage: 0.068), University of Louisville (Publications: 114, RankPage: 0.061), Jilin University (Publications: 105, RankPage: 0.057), University of Melbourne (Publications: 57, RankPage: 0.057), and University of Hong Kong (Publications: 40, RankPage: 0.346). The network is positioned in 6 clusters, each represented by Wenzhou Medicine University, University of Melbourne, University of Hong Kong, University of Auckland and Monash University, and China Medicine University.

### Contribution of authors

A total of 10,443 authors contributed to DCM-related research in the past decade, with an average of 5.53 authors per study. In Table 2, we listed the top 20 contributing authors of the DCM field in the past decade while each contributed more than 10 publications. Cai Lu was by far the most prolific author, with 41 publications and 1,480 citations, followed by Tan Yi (publications: 26, citations: 1,092) and Ritchie Rebecca H. (publications: 21, citations: 1,092), respectively, and the top 3 highest h-index authors are the same. It is noted that many scholars have recognized Lin Jie and Li Yang.

**TABLE 2:** Top 20 contributing authors of diabetic cardiomyopathy research in the past decade.

Ranking	Author	Count (% of 1887)	h-index	Total citations
1	Cai Lu	41 (2.17%)	22	1480
2	Tan Yi	26 (1.38%)	20	1092
3	Ritchie Rebecca H	21 (1.11%)	13	645
3	Zhang Wei	21 (1.11%)	13	639
4	Liang Guang	17 (0.90%)	13	631
4	Tang Qizhu	17 (0.90%)	13	576
5	Xu Changqing	16 (0.85%)	13	370
6	Li Xiaokun	15 (0.79%)	14	510
6	Zhang Yun	15 (0.79%)	12	652
7	Li Wei	14 (0.74%)	9	566
7	Sun Dongdong	14 (0.74%)	9	537
8	Wang Haichang	13 (0.69%)	10	298
8	Zheng Yang	13 (0.69%)	10	403
9	Chen Chen	12 (0.64%)	8	592
9	Chen Jing	12 (0.64%)	7	491
9	Huang Chihyang	12 (0.64%)	8	455
9	Kiriazis Helen	12 (0.64%)	10	428
9	Kuo Weiwen	12 (0.64%)	9	534
9	Lin Jie	12 (0.64%)	9	296
9	Li Yang	12 (0.64%)	9	324

**FIGURE 5:**
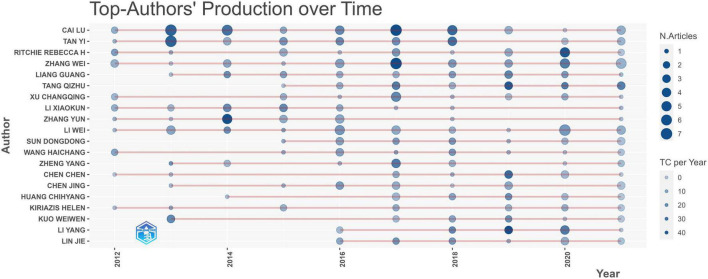
Publications and citations per year of the top 20 most prolific authors in the field of DCM research in the past decade.

The count of papers and citations per year directly indicates the author’s activity in this research field. Suppose an author produces relevant articles every year meanwhile has been cited by many other scholars. In that case, the author has been active in this field with particular influence. The result of the top 20 most prolific authors’ annual production and citations from 2012 to 2021 in the field of DCM is shown in Figure 5. It is noted that Cai Lu, Tan Yi, Ritchie Rebecca H, and Zhang Wei have been very active in DCM research with higher total citations per year.

**FIGURE 6:**
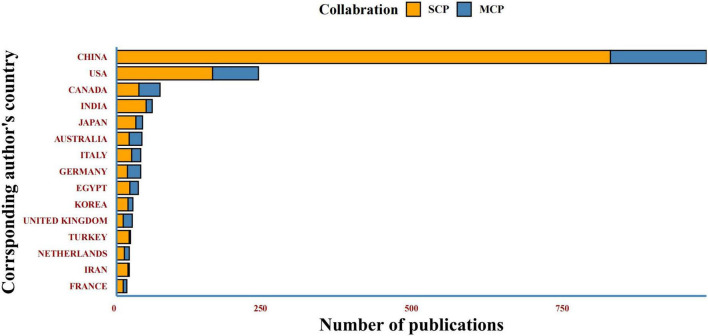
Top 20 corresponding author’s countries. SCP-Single Country Publications; MCP- Multiple Country Publications.

The top 3 corresponding author’s countries are China, the United States, and Canada, with multiple country publications ratios of 16.3%, 32.3%, and 48.6%, respectively. Most countries’ publications with co-authors involved multi-country cooperation. Among the top 20 corresponding author’s countries, Germany (18/22) and the United Kingdom (11/15) have more multiple country publications (MCP) than single country publications (SCP), while other countries of corresponding authors lack international cooperation (Figure 6).

### Outstanding journals

**FIGURE 7:**
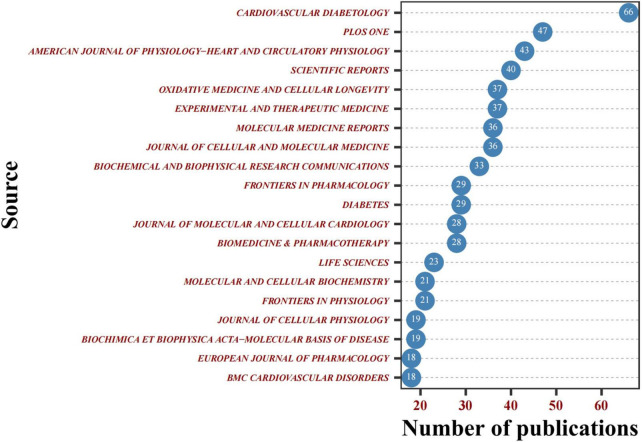
Cleveland dot pot of the top 20 journals cited journals.

In the past decade, 1,887 articles in the DCM field have been published in 459 different journals. In Table 3, the top 20 journals in terms of the count of publications are displayed, *Cardiovascular Diabetology* (*n* = 66) was the leading journal among these and with the highest impact factor (IF = 9.951), followed by *PLoS ONE* (*n* = 47) and *American Journal of Physiology-Heart and Circulatory Physiology* (*n* = 47), accounting for 2.49% and 2.28% of the overall research output respectively. The establishment of *Cardiovascular Diabetology* also indicates that diabetic cardiomyopathy is an increasingly severe diabetic complication that deserves more attention. Most of the publishers of these journals are located in the United States and England. It is worth noting that *Diabetes* (LCS = 3,244, IF = 9.461), *PLoS ONE* (LCS = 1,438, IF = 3.240), and *Cardiovascular and Diabetology* (LCS = 1,253, IF = 9.951) are also among the top 20 journals ranked by local citations (Figure 7).

**TABLE 3:** Top 20 journals contributing to publications in diabetic cardiomyopathy in the past decade.

Journal	Count (% of 1887)	Research area	Impact factor	Country/Region
Cardiovascular Diabetology	66 (3.50%)	Endocrinology and Metabolism Cardiac and Cardiovascular Systems	8.941	ENGLAND
PLoS ONE	47 (2.49%)	Multidisciplinary Sciences	3.752	United States
American Journal of Physiology-Heart and Circulatory Physiology	43 (2.28%)	Peripheral Vascular Disease Physiology Cardiac and Cardiovascular Systems	5.125	United States
Scientific Reports	40 (2.12%)	Multidisciplinary Sciences	4.996	ENGLAND
Experimental and Therapeutic Medicine	37 (1.96%)	Medicine, Research and Experimental	2.751	GREECE
Oxidative Medicine and Cellular Longevity	37 (1.96%)	Cell Biology	7.310	United States
Journal Of Cellular and Molecular Medicine	36 (1.91%)	Cell Biology Medicine, Research and Experimental	5.295	ENGLAND
Molecular Medicine Reports	36 (1.91%)	Oncology Medicine, Research and Experimental	3.423	GREECE
Biochemical And Biophysical Research Communications	33 (1.75%)	Biochemistry and Molecular Biology Biophysics	3.332	United States
Diabetes	29 (1.54%)	Endocrinology and Metabolism	9.337	United States
Frontiers In Pharmacology	29 (1.54%)	Pharmacology and Pharmacy	5.988	SWITZERLAND
Biomedicine and Pharmacotherapy	28 (1.48%)	Pharmacology and Pharmacy Medicine, Research and Experimental	7.419	FRANCE
Journal Of Molecular and Cellular Cardiology	28 (1.48%)	Cell Biology Cardiac and Cardiovascular Systems	5.763	ENGLAND
Life Sciences	23 (1.22%)	Pharmacology and Pharmacy Medicine, Research and Experimental	6.780	ENGLAND
Frontiers In Physiology	21 (1.11%)	Physiology	4.755	SWITZERLAND
Molecular And Cellular Biochemistry	21 (1.11%)	Cell Biology	3.842	NETHERLANDS
Biochimica Et Biophysica Acta-Molecular Basis of Disease	19 (1.01%)	Biochemistry and Molecular Biology Biophysics	6.633	NETHERLANDS
Journal Of Cellular Physiology	19 (1.01%)	Cell Biology Physiology	6.513	United States
BMC Cardiovascular Disorders	18 (0.95%)	Cardiac and Cardiovascular Systems	2.174	ENGLAND
European Journal of Pharmacology	18 (0.95%)	Pharmacology and Pharmacy	5.195	NETHERLANDS

### Citation analysis

**FIGURE 8:**
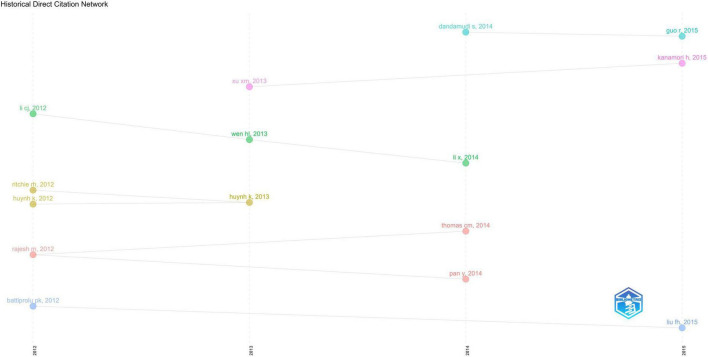
Historical analysis of direct citation of top-cited articles in DCM field from 2012–2021 generated from R studio Intellectual Structure menu of *Biblioshiny* package.

In Supplementary Table 1 (28–37), we listed the top 10 articles ranked by LCS. The higher the LCS value, the greater the influence of this article in the DCM field. Overall, as branches of the previous research, LCS were not high for articles in the past decade, which most are experimental studies of mechanisms. The top-cited paper is “Metabolic Stress-induced Activation of FoxO1 Triggers Diabetic Cardiomyopathy in Mice” (28) (LCS: 54), published in the *Journal of Clinical Investigation* (IF: 19.386 according to 2021 JCR™). We further conducted the historiographic analysis, which can provide the year-by-year mapping of the historical directly-cited publications, to better perform the citation connections among influential articles from a period. Each node represents different influential articles while lines represent their connections. Notably, articles without citation connections with others from 2012–2021 will not appear in Figure 8.

### Keywords analysis and future research direction

**FIGURE 9:**
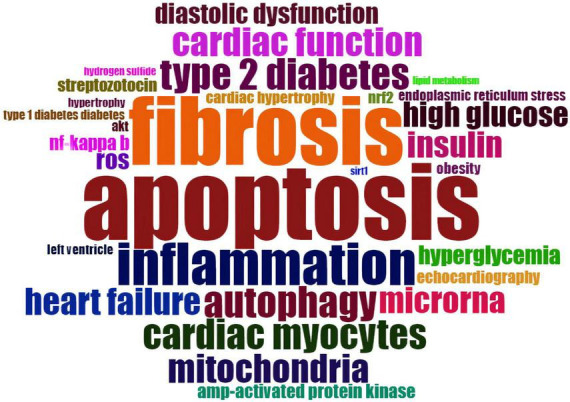
Word Cloud map regarding the keywords’ frequency of occurrence generated from R studio Documents menu of *Biblioshiny* package.

We extracted 3,147 Author’s keywords from 1,887 articles. Deleting “Diabetic cardiomyopathy” and merging synonyms, a total of 2,774 keywords were obtained, of which 31 keywords appeared more than 20 times. The frequency results are shown in Supplementary Figure 1. Then we made a Word Cloud map using the 31 Author’s keywords to understand better the current research hotspots (Figure 9).

**FIGURE 10:**
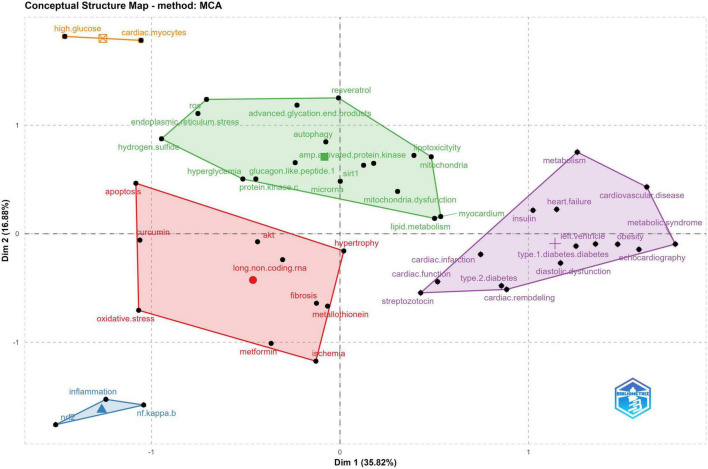
Conceptual structure map of author’s keywords based on Multiple correspondence analysis with clustering validation (5 categories identified) in DCM field generated from R studio Conceptual Structure menu of *Biblioshiny* package.

After exploring the frequency of occurrence of each keyword, we further investigated the association between them. Simple correspondence analysis (SCA) is a visual data analysis method that graphically represents the relationship between the categorical data in low dimensional space (38). Multiple correspondence analysis (MCA) is an extension of SCA. Unlike SCA, the main advantage of MCA is that it is a powerful multivariate statistical technique dealing with more than one categorical variable (39). As an unsupervised learning algorithm, this method can explore, summarize and graphically represent the association between multi-dimensional categorical data in large and complex datasets (40). In our case, MCA’s output produces points clouds of keywords typically represented by a 2-dimensional graph. The cloud of keywords is constructed on associations between keywords which can synopses the expression of relations between the articles with no underlying hypotheses. In a word, in the same quadrant, keywords with the most significant associations were located the closest. Finally, we utilized the hierarchical clustering method to cluster different clouds of keywords into five distinct categories, represented as category 1 (red), category 2 (light blue), category 3 (green), category 4 (purple), and category 5 (orange) respectively (Figure 10).

**FIGURE 11:**
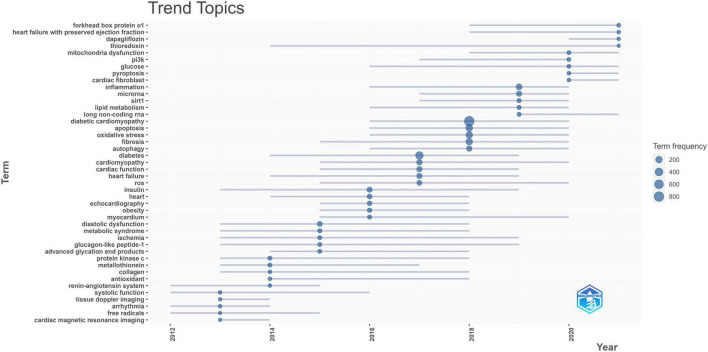
Trend topic map of author’s keywords in DCM field in the past decade generated from R studio Documents menu of *Biblioshiny* package.

The trend topic analysis is a vital mapping tool that helps to portray the seed of trend integration rooted in the previous stream (41). The topic is an induced and summarized concept, like a bucket filled with keywords with similar meanings or apparent associations. In our case, we identified the author’s keywords and examined the keywords that occur at least five times per year, and the word frequency needs to be more than five times. As shown in Figure 11, 44 buzz topics of the year were identified as. The circle represents the topic that emerged dramatically in the year, and the blue line denotes the times when the topics frequently occur. Except for the topics like “Diabetic cardiomyopathy,” “Cardiomyopathy” and “Diabetes” that we searched for, “Apoptosis,” “Oxidative stress” and “Fibrosis” have a higher frequency, “Forkhead box protein O1,” “Heart failure with preserved ejection fraction,” “Dapagliflozin,” “Thioredoxin,” “Mitochondria dysfunction,” “Glucose,” “Pyroptosis,” “Cardiac fibroblast” and “Long non-coding RNA” have potential research prospects. “Tissue Doppler imaging,” “Arrhythmia” and “Cardiac magnetic resonance imaging” as hot topics in 2013 have become less popular in the future, which may be related to the fact that they have been studied thoroughly.

## Discussion

The worldwide surge in individuals suffering from DM brings a dramatic societal burden on substantial healthcare costs and poor health outcomes for affected patients (42). Cardiovascular complications secondary to DM have aroused great concern. As one of the most common complications of DM, DCM, which have a twofold greater risk of HF compared with other complications (43), seriously affects the prognosis of diabetic patients, triggering a high mortality rate (44). Although DCM-related preclinical and clinical research have grown exponentially over the past few decades, the pathogenesis of DCM remains unclear and without consensus on preventive or therapeutic strategies to date (11). So, it is vital to sort out a practical, systematic, and comprehensive review of DCM-related papers in the past few years to enable researchers quickly understand the research status, capture the characteristics and identify the future research direction. Hence, we conducted a bibliometric analysis of 1,887 DCM-related articles from 2012–2021 (a decade) in WoSCC to provide an overview of current knowledge in various categories and potential future hotspots.

We used the *Biblioshiny* to conduct our bibliometric analysis. The chronological trends of publications show that the overall volume of annually published DCM-related original research has increased globally during the study period, which can be divided into two stages (Figure 2A). The first stage is 2012–2015, a flat period of the DCM-related original study, the number of articles fluctuated around 150. The year of 2016 is an important milestone toward the second stage when DCM research has entered rapid development and reached its highest level of 278 articles in 2021. In addition, the total mean article cited per year increased from 4.57 to 6.09 in 2019 (Figure 2B). The poor performance in 2020 may be related to the fact that the deadline for retrieval was set at the end of 2021. Therefore, the total average citations of articles in 2021 cannot be displayed either, and the mean citations of articles published in 2020 may be underestimated (19). The linear slope of publications and citations growth in the past 10 years is 0.941 and 0.341, respectively, indicating that DCM has become increasingly important as a severe pathological change in the progression of cardiovascular complications with promising research prospects.

As for global contributions, China is the most productive country with 1,232 publications. DM is a global public health problem, especially in developing countries (45, 46). As the largest developing country, China has a vast number of individuals with DM, which is still rising and underrated (47, 48). Taken together, China would emphasize more on the study of DM and its complications, including DCM. While the United States had the highest PageRank score, a novel index used to evaluate the importance of items in the collaboration map, implying that it has enormous influence with a high volume of publications as well as cooperation with many countries. Interestingly, the United Kingdom (Publications: 26, RankPage: 0.056) ranks third by PageRank value, but it has only 26 publications, much lower than other countries, which means the United Kingdom does not publish much but has a lot of connections with many other countries. Among institutions, Shandong University ranks first in the number of articles published. In terms of collaboration and influence, Wenzhou medical university, the University of Louisville, Jilin University, and the University of Melbourne have a high clustering density, showing a significant influence in the past decade in the research field of the DCM. Although the University of Hong Kong has 40 publications, it ranks fifth place according to the PageRank value, indicating it has many research partners. Relatively independent research institutions with low PageRank score need to further strengthen cooperation in the future to improve their influence.

Professor Cai Lu, from the University of Louisville in the United States, has the most significant quantity of original full-length articles (41 articles, 1,480 total citations), publishing 2% of all publications, with the greatest h-index. In the 1980s, Metallothionein (MT) was demonstrated as an antioxidant against reactive oxygen and nitrogen species (ROS, RNS) (49, 50). Later, MT was observed it could improve diabetes-induced cardiac deficits by inhibiting ROS/RNS (51). Professor Cai Lu further demonstrated that MT could impede the accumulation of ROS/RNS and prevent cardiac apoptosis by suppressing mitochondrial oxidative stress, which significantly prevents DCM development (52, 53). In general, Cai Lu has made essential contributions to DCM’s preclinical pharmacological and pathological mechanism research. Figure 5 shows that, as one of the high-yielding authors from Monash University and Baker Heart and Diabetes Institution in Australia, Ritchie Rebecca H published many papers and got more citations in the last 3 years. The therapeutic potential of cardiac-targeted gene therapy, especially the adeno-associated viral vector (AAV) gene therapy which could limit pathological remodeling in the diabetic heart and improve cardiac function, are the main research contributions of Ritchie Rebecca H (54–56). In addition, corresponding author countries are primarily domestic. However, a certain amount of international cooperative research is devoted to exploring the possible therapeutic direction of DCM. Researchers from the United Arab Emirates, the United States, Lebanon, Saudi Arabia, and Australia have discussed the progress made in the mechanism of phytochemicals’ cardioprotective effect in DM (57), meaning the treatment of DCM is a topic of global interest and consensus, and the research on the treatment of DCM with traditional Chinese medicine may have promising development potential.

Although Asian countries like China lead the area with the most counts of DCM-related articles in the past decade, most of these journals’ publishers are located in United States and England. It suggested that Asian countries should strengthen the construction of journals. *Cardiovascular Diabetology* journal released the most DCM-related articles with a high IF, while the *Diabetes* journal had the highest IF according to the 2021 JCR™. Both journals appeared in the top 20 journals regarding LCS (Figure 7), reflecting that these two journals have some authority in the DCM research field.

Despite the fact that the article published in 2012 by Battiprolu PK et al. has not been cited by other high-quality papers. The most significant article with the highest LCS in the past decade has not been cited by the majority of classically-cited articles. A possible explanation is that this article was cited by other newly published papers which have not yet shown their academic impact (high LCS) to date, indicating that it may have a promising research prospect. This study demonstrated that the Forkhead box proteins O (FoxO) signaling axis was persistently activated in DCM development for the first time (28). Interestingly, we also found that FoxO1 has enormous developmental potential in our future trend topics analysis (Figure 11). Unlike other bibliometric studies (19, 58), our historical analysis does not reflect the inheritance relationship between classically-cited literature, which may be related to the selected research period. Figure 7 may show the branches of previous studies since DCM research has entered a period of rapid development in the past decade.

We can track the knowledge distribution, association, and future research directions by analyzing the author’s keywords which are a scientific article’s most concise and accurate generalization. We conducted the analysis from two dimensions. The first dimension is we explored the frequency of keywords and the possible association between them from a cross-sectional perspective. The top three keywords in frequency in the past decade are apoptosis (216 times), oxidative stress (213 times), and fibrosis (192 times), respectively (Figure 9). Diabetes is characterized by oxidative stress and low-grade inflammation (59). Under physiological circumstances, a delicate balance between the production and degradation of ROS resulting an average steady-state ROS level. as a predilection site of oxidative stress, the myocardium is vulnerable to a transient or persistent ROS increment due to the DM, which would result in oxidative modification of cellular component and eventually induce cell death *via* apoptosis (60).

It is worth interpreting the significance of the coordinate axes in Figure 10. The first coordinate axis (x-axis) emphasizes the characteristics of the DCM study. Higher values (at the right of the conceptual structure map) are concepts related to metabolic, functional, and organ level changes, such as obesity, left ventricle, heart failure, etc. These factors are undoubtedly hot issues in clinical research in the field of DCM because the change in cardiac function and Metabolic problems such as obesity can be observed directly. Conversely, lower values for the x-axis indicate the pathological study in the DCM field. These paramount results say that different characteristics of the DCM study at the bench and clinical courses exist. The second coordinate axis (y-axis) can be interpreted as the studies focusing on some hot issues on the dynamics (61). Overall, the hotspots of increasing interest in DCM research in the last decade mainly focused on pathological mechanisms at the molecular and cellular level.

Considering the findings from MCA results, the interpretation of the Cluster analysis results is that Category 1, Category 2, and Category 3 mainly focus on the DCM study of pathological mechanisms, including autophagy, apoptosis, fibrosis, signaling pathways, etc. Category 4 represents the pathophysiology mechanisms and clinical manifestation of DCM. Interestingly, Category 5 depicts the effects of the hyperglycemic effect on cardiomyocytes with only two items. As endogenous non-coding RNA (ncRNA) molecules, microRNAs (miRNA) can significantly affect different biological processes, primarily through the suppression of mRNA expression. However, the synthesis of those molecules is affected by high glucose levels (62). A recent study found that more than 300 miRNAs are dysregulated in DCM (63), which modulate plenty of cardiomyocyte pathophysiological processes, including apoptosis, inflammation, cell growth, pyroptosis, fibrosis, and response to oxidative stress *via* different pathways (64–67). Although there are many different levels of understanding of the mechanism of DCM, similar to previous studies, hyperglycemia seems to play an essential role in the pathogenesis of diabetic cardiomyopathy, activating a series of pathological changes or processes (68).

The second dimension shows the research trend of DCM in the past 10 years in combination with the evolution of time. Overall, these current and promising future research frontiers reflect that the pathological changes of DCM are diverse. However, the research topics with promising research potential in the future are gradually transitioning to drug-based treatment (Figure 11). As discussed above as well as in previous reviews, metabolic disturbance, including hyperglycemia, is a central and essential driver of pathological changes (oxidative stress, cardiac hypertrophy and fibrosis, inflammation, apoptosis, etc.) modulated by a wide range of molecules and cells, affecting the structure and function of the cardiac, particularly the left ventricle (4, 69). Although a few studies have addressed various pathological alterations, the causal relationship between these complex molecular and cellular mechanisms has not been fully elucidated (70). It has ultimately led to the conclusion that there is currently no standard pharmacotherapeutic approach for DCM. Even though diagnostic criteria for pure DCM are demanding, several drugs still showed the treatment potential, inspiring future drug-based therapeutic clinical studies [Supplementary Table 2 (71–77)].

The new class of antidiabetic drugs Dapagliflozin (DAPA) is a member of sodium-glucose cotransporter-2 (SGLT-2) inhibitors showing promising benefits on DM individuals with CVD (78). DAPA has been proved that it could suppress cardiac fibroblast activation and endothelial-to-mesenchymal transition (EndMT) to protect against myocardial fibrosis *via* AMP-activated protein kinaseα (AMPKα)-modulated inhibition of TGF-β/Smad signaling (79). Previous studies have reported that DAPA could also protect cardiomyocytes from inflammation and oxidative stress damage or up-regulating erythropoietin (EPO) levels to decrease apoptosis (80–82). The thioredoxin system is a ubiquitous family of cysteine-dependent antioxidant proteins with a robust ROS scavenging capacity in the cardiomyocyte antioxidant network (83). Overexpression of thioredoxin has been demonstrated to have a momentous functional implication in mitigating cardiomyocyte dysfunction from DM-induced oxidative stress, which is expected to become a key target for the drug-based treatment of DCM (84, 85).

As the most widely studied subtype of the FoxO family, FoxO1 is commonly involved in the regulation of cell metabolism, apoptosis, and differentiation, especially in pancreatic β-cell (86–88). FoxO1 is a critical transcription factor in insulin cascade affected by different upstream signaling molecules [e.g., phosphatidylinositol 3-kinase (PI3K)/Akt, AMP-activated protein kinase (AMPK), and Sirtuin 1 (SIRT 1)], and it can regulate several downstream proteins, including myocardial pyruvate dehydrogenase (PDH) and peroxisome proliferator-activated receptor α (PPARγ) coactivator-1α (PGC-1α) (89–92). In 2021, several studies proved inhibition of FoxO1 may be an approach to alleviate cardiac fibrosis, diastolic dysfunction, and left ventricular dysfunction and remodeling in the mouse model of DCM (93–95). Thus, FoxO1 could be an attractive target for the pharmacotherapy of DCM.

In addition, ncRNAs have recently played a worthy note regulatory role in human health and disease (96). As one of the most promising topics in the field of DCM, long ncRNAs (LncRNAs) can actively participate in the pathogenesis of CVD, including DCM especially (97). Moreover, like sponges, LncRNAs can block the regulatory function of miRNAs by binding to miRNAs and hindering the interaction with their target (98). Several studies suggested that LncRNAs could mediate cardiomyocyte apoptosis induced by high glucose (99, 100), regulate cardiac remodeling *via* the TGF-β/Smads pathway (101, 102), and mediate cardiomyocyte injury (e.g., ischemia-reperfusion damage, lipotoxic injury) (103, 104).

Regarding clinical significance, our research results indicate that the phenotype of heart failure with preserved ejection fraction (HFpEF) in DCM is attracting more attention, which is discussed above as a precursor stage of heart failure with reduced ejection fraction (HFrEF) phenotype. However, there are also studies indicating that clinical DCM is a two-sided disease composed of HFpEF and HFrEF independently with distinct myocardial effects (10). The occurrence of HFpEF in DCM is primarily due to left ventricular diastolic dysfunction (LVDD) through increased cardiomyocyte stiffness and hypertrophy with high resting tension, which manifests as the left ventricular ejection fraction (LVEF) being greater than or equal to 50% (7, 105). Similar hemodynamic effects were also observed in large clinical studies with a broader range of diabetic patients, to which cardiovascular risk factors including CVD may contribute (106, 107). Thus, the mechanism studies of LVDD in DCM have the potential to provide important insight into DM augmentation on HFpEF. Microcirculation rarefaction and AGES microvascular deposition were observed in HFpEF and HFrEF. However, hyperglycemia, insulin resistance, and lipotoxicity are more closely related to HFpEF, whereas inflammation and autoimmune response are more closely related to HFrEF (108). DCM was proposed and defined over 50 years ago, which characteristics make it challenging to conduct relevant clinical research. However, its enlightening significance was not limited to cardiac dysfunction without CVD and HTN in individuals with DM but expanded to describe the increased vulnerability of the myocardium to metabolism dysfunction of diabetic patients when DM acts like a sole perpetrator (109). In other words, the significance of the study of DCM is to explore the direct effect of glucose-related metabolism disorder on cardiac function independently of other cardiovascular risk factors, which aims to alleviate diabetes augmentation on cardiovascular disease and further improve the symptoms and prognosis of cardiovascular complications of DM.

Our research has two common limitations of bibliometrics (19, 21). One limitation is that only the WoSCC database was selected for publications search due to the R package bibliometrix could not combine other databases to date. However, we are confident that most authority and DCM-related publications were retrieved from the WoSCC database, considered one of the top sources for bibliometric analysis with a well-established citation network (18). The other one is we only included original full-length articles. Non-article publications, including reviews, were excluded from our study, which may have ignored theoretical research hotspots.

## Conclusion

We conducted a bibliometric analysis to comprehensively perform the current state of the DCM knowledge domain from 2011 to 2021. Overall, the annual quantity of published articles has increased steadily. China and the United States were found to be influential in this field. Shandong University, professor Cai Lu, and the *Cardiovascular and Diabetology* journal has the highest volume of articles in terms of institutions, authors, and journals. The cooperation between authors, institutions, and countries should be further strengthened in the future. The mainstream DCM research field mainly focused on pathological mechanisms, including apoptosis, oxidative stress, and fibrosis at the molecular and cellular level, and gradually delved into mechanisms studies of DCM drug-based treatment. We believe our bibliometric-based study will benefit academics who focus their work on hotspots reducing outdated research through a comprehensive developed framework.

## Data availability statement

The original contributions presented in this study are included in the article/Supplementary material, further inquiries can be directed to the corresponding author/s.

## Author contributions

SW conceived the study and wrote the first draft of the manuscript. CT helped to draft the manuscript. ZG helped to carry out data analysis and polish the manuscript. BZ and LZ provided the critical revisions. All authors revised the manuscript and approved the submitted version.

## Footnotes

1
http://isiknowledge.com/

2
http://mjl.clarivate.com/
