# Supplementary material for: Research status and trends of the diabetic cardiomyopathy in the past 10 years (2012–2021): A bibliometric analysis
Source: Front Cardiovasc Med. 2022 Oct 20;9:1018841. doi: 10.3389/fcvm.2022.1018841 (PMC9630656; doi:10.3389/fcvm.2022.1018841)
Supplement: Supplementary file 1 [file Data_Sheet_1.docx]

Supplementary Material

# Supplementary Figures and Tables

## Supplementary Figure

**
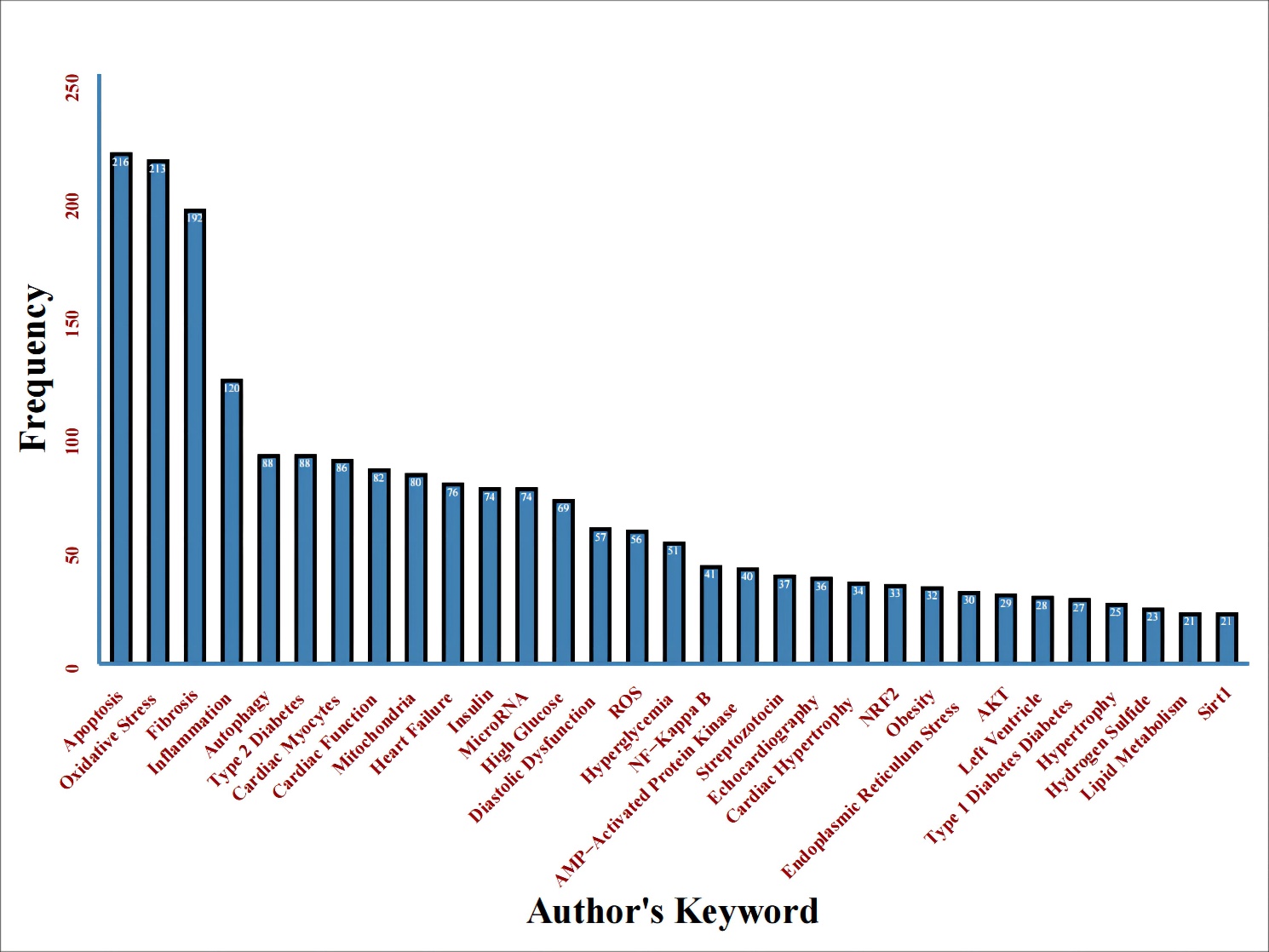
**

**Supplementary Figure 1.** Author’s keywords that appeared more than 20 times ranked by frequency of occurrence

## Supplementary Table

**Supplementary Table 1.** Top 10 well-cited articles ranked by LCS

| **Authors** | **Title** | **DOI** | **Year** | **LCS** | **Subcategory** | **Sources** |
| --- | --- | --- | --- | --- | --- | --- |
| Battiprolu PK, et al. (28) | Metabolic Stress-induced Activation of FoxO1 Triggers Diabetic Cardiomyopathy in Mice. | 10.1172/JCI60329 | 2012 | 54 | Experimental study | *Journal of Clinical Investigation* |
| Li CJ, et al. (29) | Cardiac Fibrosis and Dysfunction in Experimental Diabetic Cardiomyopathy are Ameliorated by Alpha-lipoic Acid. | 10.1186/1475-2840-11-73 | 2012 | 51 | Experimental study | *Cardiovascular Diabetology* |
| Huynh K, et al. (30) | Targeting the Upregulation of Reactive Oxygen Species Subsequent to Hyperglycemia Prevents Type 1 Diabetic Cardiomyopathy in Mice. | 10.1016/j.freeradbiomed.2013.02.021 | 2013 | 45 | Experimental study | *Free Radical Biology and Medicine* |
| Pan Y, et al. (31) | Inhibition of JNK Phosphorylation by a Novel Curcumin Analog Prevents High Glucose–Induced Inflammation and Apoptosis in Cardiomyocytes and the Development of Diabetic Cardiomyopathy. | 10.2337/db13-1577 | 2014 | 45 | Experimental study | *Diabetes* |
| Bai Y, et al. (32) | Prevention by Sulforaphane of Diabetic Cardiomyopathy is Associated with Up-regulation of Nrf2 Expression and Transcription Activation. | 10.1016/j.yjmcc.2013.01.008 | 2013 | 43 | Experimental study | *Journal of Molecular and Cellular Cardiology* |
| Rajes, et al. (33) | Cannabinoid 1 Receptor Promotes Cardiac Dysfunction, Oxidative Stress, Inflammation, and Fibrosis in Diabetic Cardiomyopathy. | 10.2337/db11-0477 | 2012 | 42 | Experimental study | *Diabetes* |
| Kanamo H, et al. (34) | Autophagic Adaptations in Diabetic Cardiomyopathy Differ Between Type 1 and Type 2 Diabetes. | 10.1080/15548627.2015.1051295 | 2015 | 39 | Experimental study | *Autophagy* |
| Li X, 2014, et al. (35) | MicroRNA-30d Regulates Cardiomyocyte Pyroptosis by Directly Targeting Foxo3a in Diabetic cardiomyopathy. | 10.1038/cddis.2014.430 | 2014 | 31 | Experimental study | *Cell Death & Disease* |
| Xu XM, et al. (36) | Diminished Autophagy Limits Cardiac Injury in Mouse Models of Type 1 Diabetes. | 10.1074/jbc.M113.474650 | 2013 | 30 | Experimental study | *Journal of Biological Chemistry* |
| Dandamudi S, et al. (37) | The Prevalence of Diabetic Cardiomyopathy: A Population-Based Study in Olmsted County, Minnesota. | 10.1016/j.cardfail.2014.02.007 | 2014 | 30 | Epidemiological study | *Journal of Cardiac Failure* |

**Supplementary Table 2.** DCM-related clinical studies based on published articles

| **Role** | **Category of drug** | **Drug** | **Exclusion criteria** | **Conclusions** | **References (first author-year)** |
| --- | --- | --- | --- | --- | --- |
| Improve metabolic disorders | Glucagon-like peptide-1 receptor agonist | Liraglutide | History or presence of renal, hepatic or cardiovascular disease, etc. | Liraglutide alleviated the load on the left ventricle by reducing early left ventricular diastolic filling and left ventricular filling pressure. | Bizino MB-2018. (71) |
|  | Cu (II)-selective chelator | Trientine | History or evidence of significant cardiac valvular disease, autonomic neuropathy, cardiac valvular disease, left ventricular wall motion abnormality, etc. | Trientine significantly reduced left ventricular mass index to normal levels without causing adverse remodeling. | Cooper GJS-2009. (72) |
|  | Nuclear receptor peroxisome proliferator-activated receptor gamma agonists | Rosiglitazone | Valvular heart disease, rhythm other than normal sinus rhythm, left ventricular systolic dysfunction, known coronary artery disease, suspicion of clinical coronary artery disease, renal and liver dysfunction, etc. | Rosiglitazone and pioglitazone improved diastolic function, but only rosiglitazone significantly improved systolic myocardial function. | Pala S-2010. (73) |
|  |  | Pioglitazone | Any clinically significant disorder, particularly any history or complaints of cardiovascular or liver disease or diabetes-related complications. | Pioglitazone was associated with improved measures of left ventricular diastolic function, myocardial glucose uptake, and whole-body insulin sensitivity. | van der Meer RW-2009. (74) |
|  | Sulfonylureas | Gliclazide | Severe valvular heart disease, atrial fibrillation, uncontrolled blood pressure, symptoms or signs or history of cardiovascular disease, etc. | Compared with glibenclamide, a significant decrease in LV mass index was observed in the gliclazide group after six months of treatment. | Lee TM-2007. (75) |
| Anti-remodeling | Phosphodiesterase type 5 inhibition | Sildenafil | History of cardiovascular disease, autonomic neuropathy, symptoms or signs of ischemic heart disease during cardiac evaluations at enrollment, etc. | Sildenafil ameliorated left ventricular hypertrophy in DCM, independent of other vasodilatory or endothelial effects. | Giannetta E-2012. (76) |
|  | Dipeptidyl peptidase-4 inhibitors | Sitagliptin | Severe heart failure, respiratory failure, uncontrolled hypertension, coronary heart disease, arrhythmias, etc. | Sitagliptin had cardioprotective effects independent of glucose control. | Nogueira KC-2014. (77) |
